# Substance Use Treatment Services in New York (2021–2023): A State Profile Analysis Based on National Survey of Substance Abuse and Mental Health Services (N-SUMHSS) Data

**DOI:** 10.7759/cureus.84029

**Published:** 2025-05-13

**Authors:** Abimbola E Arisoyin, Esther I Ezeani, Edediong Ekarika, Amaka S Odega, Oscar O Ahumaraeze, Okelue E Okobi

**Affiliations:** 1 Psychiatry, Harlem Hospital Center, New York, USA; 2 Family Medicine, Indiana Regional Medical Center (IRMC), Indiana, USA; 3 Primary Care, Lifebridge Health, Baltimore, USA; 4 Public Health, Emory University Rollins School of Public Health, Atlanta, USA; 5 Medicine, All Saints University School of Medicine, Roseau, DMA; 6 Psychiatry, Northern Ontario School of Medicine (NOSM) University, Sault Ste. Marie, CAN; 7 Pediatric Ophthalmology, All Saints University School of Medicine, Roseau, DMA; 8 Family Medicine, IMG Research Academy and Consulting LLC, Homestead, USA; 9 Family Medicine, Larkin Community Hospital Palm Springs Campus, Hialeah, USA; 10 Family Medicine, Lakeside Medical Center, Belle Glade, USA

**Keywords:** new york city, n-sumhss data, retrospective analysis, substance use disorder (sud), treatment services

## Abstract

Background: Substance use disorders (SUDs) are a significant public health issue in the U.S., with New York State facing rising treatment demands. The National Survey of Substance Abuse and Mental Health Services (N-SUMHSS) offers valuable data on treatment trends across the state.

Objective: This study analyzes trends in substance use treatment services in New York from 2021 to 2023 using N-SUMHSS data, focusing on facility capacity, medication-assisted treatment (MAT) utilization, and treatment effectiveness.

Methods: Secondary data from the N-SUMHSS (2021-2023) were analyzed, examining facility types, client numbers, and MAT use (methadone, buprenorphine, naltrexone). Descriptive statistics and trend analysis were conducted to assess changes in treatment services.

Results: From 2021 to 2023, the number of facilities remained stable, with slight fluctuations. Private non-profit organizations dominated, comprising 69.7% of facilities in 2022-2023. MAT utilization declined in both opioid treatment programs (OTPs) and non-OTP facilities, with methadone usage remaining prevalent. Clients receiving MAT at OTP facilities decreased from 39,534 (9.3%) in 2021 to 34,186 (7.8%) in 2023, while naltrexone use rose. Outpatient services increased to 547 (72.1%), while residential and hospital inpatient facilities saw slight declines.

Conclusion: MAT utilization has improved, but challenges persist in addressing the growing demand for residential and detox services. Expanding outpatient and inpatient services, along with greater access to MAT, is crucial to improving treatment in New York.

## Introduction

Substance use disorders (SUDs) represent a persistent and growing public health concern, profoundly impacting individuals, families, and communities. The complexity of SUDs requires a multifaceted approach encompassing prevention, treatment, and recovery support services [1]. Effective treatment options, including behavioral therapies and medication-assisted treatment (MAT), are crucial in reducing the burden of SUDs and improving patient outcomes. Understanding trends in treatment availability, facility operations, and therapeutic approaches is essential to addressing gaps in care and enhancing access to quality services [1,2]. Strengthening the infrastructure for SUD treatment can play a pivotal role in mitigating its societal and economic impacts [1-3].

Substance use remains a significant issue in New York, with alcohol and drugs leading to high treatment admissions [4]. Globally, alcohol use disorder accounts for 95 million cases, and illicit drug use affects 250 million, with 29 million facing disorders. Alcohol and drug use contribute substantially to the global burden of disease, measured in disability-adjusted life years (DALYs). In 2000, alcohol and tobacco each accounted for 4% of the global burden, while illicit drugs contributed 0.8%. By 2016, drug use was responsible for 31.8 million DALYs, and alcohol for 99.2 million DALYs, highlighting the severe impact on health [1,5].

SUDs are characterized by the compulsive use of alcohol or drugs despite negative consequences [6]. Prolonged substance use can lead to structural and functional changes in the brain, particularly in areas governing reward, decision-making, and impulse control. These changes perpetuate a cycle of dependence and withdrawal, making recovery challenging. The high prevalence of SUDs in New York underscores the relevance of accessible, evidence-based treatment strategies, including medication-assisted treatment (MAT), cognitive-behavioral therapy (CBT), and contingency management, to address both the physiological and psychological aspects of addiction [7,8].

This study relies on data from the N-SUMHSS, a comprehensive survey designed to capture detailed information on substance use treatment services across the United States. The database provides insights into facility operations, client demographics, treatment modalities, and capacity utilization. For the years 2021-2023, it offers a robust foundation for analyzing trends in substance use treatment in New York, identifying gaps in services, and evaluating the impact of policy changes over time [9].

The primary aim of this study is to analyze the availability, utilization, and outcomes of substance use treatment services in New York. Using N-SUMHSS data from 2021-2023, this study will (i) identify key trends in facility operations, (ii) assess client demographics and treatment types, and (iii) highlight areas for improvement to optimize care delivery. This study seeks to support evidence-based decision-making for policymakers, healthcare providers, and stakeholders involved in substance use treatment.

## Materials and methods

Data source and study design

This study is a descriptive analysis based on secondary data extracted from the N-SUMHSS for the years 2021, 2022, and 2023. N-SUMHSS is a comprehensive database that provides detailed insights into substance use treatment services across the United States, including facility operations, treatment modalities, and client demographics. The study design focuses on profiling substance use treatment services in New York State, leveraging facility-level data and treatment trends to identify key operational metrics and service patterns.

Study participants and questionnaires

The units of analysis were substance use treatment facilities in New York State that reported data to N-SUMHSS between 2021 and 2023. These facilities provide services for a variety of substance use disorders, including opioid use disorder (OUD) and alcohol dependence. Data was collected using standardized questionnaires covering facility operations, treatment modalities, client demographics, and therapeutic approaches.

Data collection and quality assurance

Data for this study were drawn from the National Survey of Substance Abuse and Mental Health Services (N‑SUMHSS) annual facility surveys for 2021-2023, with our sampling frame comprising all licensed substance use treatment providers in New York State, including outpatient, residential, and hospital‑based programs, that reported at least nine months of data in any calendar year (inclusion criterion). The standardized questionnaire captured facility characteristics (ownership type, service mix, designated bed capacity), client demographics, and treatment modalities (methadone, buprenorphine, naltrexone availability; therapeutic and telehealth approaches). All data were processed and analyzed with IBM SPSS Statistics for Windows, Version 27 (Released 2020; IBM Corp., Armonk, New York, United States) using descriptive statistics and year‑over‑year percentage change calculations (analytical technique; no inferential testing performed). To ensure data reliability, N‑SUMHSS applied automated validity checks to flag implausible values, cross‑verified outlier responses against prior survey returns and state licensing records, and conducted follow‑up queries to resolve missing or conflicting entries; data collection adhered to uniform definitions and protocols. Nonetheless, because the information is self‑reported, there remains potential for reporting bias and variability in completeness across facilities, which we have carefully considered in our descriptive analyses.

Variables of interest

The variables of interest in this study include a range of factors that contribute to the overall assessment of substance use treatment services. Facility Operation encompasses ownership types such as private for-profit, private non-profit, state government, local/county/community government, tribal government, and federal government. Clients in Treatment for Substance Use Disorders involves total client counts, demographics such as age groups, and population-adjusted treatment rates to understand the breadth and reach of services. Facility Capacity and Utilization Rate focuses on the number of facilities, designated beds, and utilization rates, particularly for residential and hospital inpatient services. Facilities Providing Medications for Opioid Use Disorder (MOUD) details the facilities offering methadone, buprenorphine, and naltrexone, and the number of clients utilizing these treatments. Clients receiving MOUD at Opioid Treatment Program (OTP) Facilities tracks clients receiving MAT at OTP-certified and non-OTP-certified facilities, expressed in rates per 100,000 population. Type of Care includes facility-level data on various service types, such as outpatient, residential, and inpatient care. Finally, Clinical/Therapeutic Approaches Used Frequently provides insights into the evidence-based therapeutic practices employed, including cognitive-behavioral therapy, motivational interviewing, relapse prevention, and telehealth services, crucial for assessing the quality and efficacy of treatment.

Data analysis and statistical methods

Descriptive statistics were used to summarize the data, including frequencies, percentages, and medians. Facility-level data and client demographics were analyzed to identify trends and patterns over the three-year period. Comparative analysis was conducted using year‑over‑year percentage change to descriptively assess trends in capacity utilization, MOUD provision, and therapeutic approaches without applying inferential statistical tests. Data was analyzed using SPSS statistical software, ensuring accurate and efficient processing.

Ethical considerations

As this study utilized publicly available de-identified secondary data from N-SUMHSS, it did not require ethical approval or informed consent. However, all data handling adhered to ethical standards for confidentiality and integrity in research. The findings aim to inform policy and service improvements without compromising the privacy of facilities or clients.

## Results

Trends in facility operations

The results from the N-SUMHSS data analysis reveal significant trends in facility operations, client treatment demographics, and the utilization of substance use treatment services across New York State from 2021 to 2023. The operation of substance use disorder treatment facilities has seen notable shifts over the past three years, with variations in both the number of facilities and the types of clients served. Private non-profit organizations have consistently dominated the landscape, comprising 544 (70.6%) of facilities in 2021, with a slight decrease to 576 (69.7%) in 2022 and 529 (69.7%) in 2023. These organizations also served the majority of clients, accounting for 88,643 (74.8%) in 2021, dropping to 82,422 (69.9%) in 2022, and further declining to 67,224 (66.8%) in 2023. On the other hand, private for-profit organizations have shown an increase in both the number of facilities and clients served, from 148 (19.2%) of facilities and 18,729 (15.8%) of clients in 2021 to 163 (19.7%) of facilities and 22,528 (22.4%) of clients in 2023. Table 1 shows the distribution of facility types, total clients, and clients under 18 across different operational models from 2021 to 2023.

**Table 1:** Trends in facility operations and client demographics n (%): Number (percentage); — (-): Data not available * represents the suppressed value (% lower than 0.0005)

Facility Type	Facilities			All Clients			Clients Under 18		
	2021 n(%)	2022 n(%)	2023 n(%)	2021 n(%)	2022 n(%)	2023 n(%)	2021 n(%)	2022 n(%)	2023 n(%)
Private for-profit organization	148 (19.2%)	163 (19.7%)	149 (19.6%)	18,729 (15.8%)	25,009 (21.2%)	22,528 (22.4%)	124 (3.7%)	159 (6.0%)	101 (5.1%)
Private non-profit organization	544 (70.6%)	576 (69.7%)	529 (69.7%)	88,643 (74.8%)	82,422 (69.9%)	67,224 (66.8%)	3,193 (94.7%)	2,368 (88.9%)	1,577 (79.9%)
State government	21 (2.7%)	26 (3.1%)	23 (3.0%)	1,782 (1.5%)	2,202 (1.9%)	2,040 (2.0%)	2 (0.1%)	— (-)	1 (0.1%)
Local, county, or community government	43 (5.6%)	46 (5.6%)	43 (5.7%)	7,420 (6.3%)	7,081 (6.0%)	6,782 (6.7%)	52 (1.5%)	133 (5.0%)	238 (12.1%)
Tribal government	4 (0.5%)	4 (0.5%)	4 (0.5%)	56 (*)	35 (*)	88 (0.1%)	2 (0.1%)	3 (0.1%)	— (-)
Federal government	11 (1.4%)	11 (1.3%)	10 (1.3%)	1,814 (1.5%)	1,182 (1.0%)	1,907 (1.9%)	— (-)	— (-)	57 (2.9%)
Total	771 (100%)	826 (100%)	759 (100%)	118,444 (100%)	117,931 (100%)	100,569 (100%)	3,373 (100%)	2,663 (100%)	1,974 (100%)

State, local, and tribal government facilities have remained relatively stable, serving a smaller proportion of clients overall. State government facilities served 1,782 (1.5%) of clients in 2021, increasing to 2,040 (2.0%) by 2023, while local government facilities served between 7,420 (6.3%), 7,081 (6.0%) and 6,782 (6.7%) of clients over the three years. Tribal government facilities have remained marginal, with a minor increase in clients served by 2,023 (88, 0.1%). Federal government facilities have seen fluctuations, serving 1,814 (1.5%) of clients in 2021, dropping to 1,182 (1.0%) in 2022, and rising to 1,907 (1.9%) in 2023.

A key trend from the data was the proportion of clients under 18 years old. Private non-profit organizations have historically served most younger clients, but their share has decreased from 3,193 (94.7%) in 2021 to 1,577 (79.9%) in 2023. Conversely, local, county, or community government facilities saw a significant increase in younger clients, from 52 (1.5%) in 2021 to 238 (12.1%) in 2023. Federal government facilities, which served no clients under 18 in 2021 and 2022, began to serve a small number of younger clients in 2023, accounting for 57 (2.9%) of the total. This data reflects shifting trends in facility operations, indicating both a diversification of facility types and the changing demographics of clients served.

Trends in substance use disorder treatment

From 2021 to 2023, facilities treating alcohol and other substances consistently represented the majority, declining slightly from 585 (92.6%) in 2021 to 555 (90.8%) in 2023. Clients receiving treatment for both alcohol and substances decreased from 47,903 (41.5%) in 2021 to 37,902 (37.9%) in 2023, while clients treated for only alcohol showed a relative increase, from 15,968 (13.8%) in 2021 to 18,778 (18.8%) in 2023. Similarly, facilities treating only alcohol consistently represented approximately 75-76% across the years. Table 2 presents the distribution of facilities, total clients, clients aged 18+, and their respective rates per 100,000 population by substance type and year from 2021 to 2023.

**Table 2:** Clients in treatment for substance use disorders by substance type, year, and demographic characteristics n (%): Number (percentage)

Substance Type	Year	Both Alcohol and Substances Other Than Alcohol	Only Alcohol	Only Substances Other Than Alcohol	Total
Facilities (n (%))	2021	585 (92.6%)	477 (75.5%)	539 (85%)	632 (100%)
	2022	614 (91.6%)	505 (75.4%)	583 (81.3%)	669 (100%)
	2023	555 (90.8%)	465 (76.1%)	542 (88.7%)	611 (100%)
All Clients (n (%))	2021	47,903 (41.5%)	15,968 (13.8%)	51,551 (44.7%)	115,422 (100%)
	2022	41,727 (36.5%)	18,198 (15.9%)	54,303 (47.5%)	114,228 (100%)
	2023	37,902 (37.9%)	18,778 (18.8%)	43,445 (43.4%)	100,125 (100%)
Clients per 100,000 Population	2021	241	81	260	582
	2022	212	92	276	581
	2023	194	66	222	512
Clients Aged 18+ (n (%))	2021	46,703 (41.8%)	15,330 (13.7%)	49,694 (44.5%)	111,727 (100%)
	2022	40,594 (36.4%)	17,549 (15.7%)	53,445 (47.9%)	111,588 (100%)
	2023	36,606 (37.6%)	18,043 (18.5%)	42,787 (43.9%)	97,436 (100%)
Clients per 100,000 Population (18+ years)	2021	297	98	316	711
	2022	259	112	341	741
	2023	234	116	274	624

Clients per 100,000 population followed a declining trend for all substance categories. For example, those treated for only alcohol decreased from 81 in 2021 to 66 in 2023, and for clients aged 18+, a similar decline was observed, with rates for both alcohol and other substances falling from 297 in 2021 to 234 in 2023.

Trends in facility capacity and utilization

From 2021 to 2023, residential facilities saw variations in capacity and utilization. The number of residential facilities decreased from 152 (2021) to 142 (2023), while the utilization rate increased from 3,818 (77.9%) (clients utilizing 4,899 designated beds) in 2021 to 4,647 (83.7%) (clients utilizing 5,552 beds) in 2023. The average number of beds per facility grew from 32 in 2021 to 39 in 2023. Table 3 summarizes the number of facilities, clients, designated beds, utilization rates, and average beds per facility for residential and hospital inpatient services from 2021 to 2023.

**Table 3:** Facility capacity and utilization rates for residential and hospital inpatient services n: Number; %: Percentage; - : No data available

Attribute	Year	Number of Facilities (n)	Number of Clients (n)	Number of Designated Beds (n)	Utilization Rate (%)	Avg. Beds per Facility
Residential (2021)	2021	152	3,818	4,899	77.9	32
	2022	197	5,564	7,071	78.7	36
	2023	142	4,647	5,552	83.7	39
Hospital Inpatient (2021)	2021	59	1,453	1,907	76	32
	2022	63	2,210	2,360	93	37
	2023	69	3,031	2,486	121	36

Hospital inpatient services showed a marked rise in utilization rates, increasing from 76% (1,453 clients utilizing 1,907 designated beds) in 2021 to 3,031 (121%) (clients utilizing 2,486 beds) in 2023, reflecting a significant demand for these services. The number of facilities increased from 59 in 2021 to 69 in 2023, while the average beds per facility remained consistent, ranging from 32 in 2021 to 37 in 2022 and 36 in 2023. The number of clients served in hospital inpatient settings more than doubled, from 1,453 in 2021 to 3,031 in 2023, highlighting the growing reliance on inpatient care.

Trends in the type of care utilization

**Figure 1:**
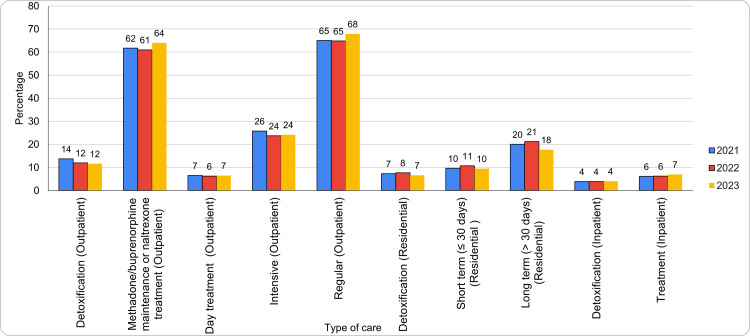
Trends in type of care utilization

Outpatient care remained the most commonly provided type of care, with facilities increasing from 549 in 2021 (71%) to 579 in 2022 (70.1%), serving 547 (72.1%) clients in 2023. Outpatient detoxification services showed a decline, decreasing from 106 (13.7%) in 2021 to 99 (12%) facilities in 2022, and further to 88 (11.6%) facilities in 2023. Similarly, outpatient methadone/buprenorphine maintenance or naltrexone treatment facilities rose from 476 (62%) in 2021 to 504 (61%) in 2022, serving 486 (64%) clients in 2023. Figure 1 summarizes the trends in type of care utilization. The types of care encompass outpatient, residential (non-hospital), and hospital inpatient services, including detoxification, MAT, intensive therapy, and varying durations of residential or inpatient treatment tailored to patient needs.

Day treatment remained stable at 50 (7%) in 2021, 51 (6.2%) in 2022, and 49 (6.5%) in 2023. Intensive outpatient declined from 199 (26%) in 2021 to 184 (24.2%) in 2023. Regular outpatient increased from 501 (65%) in 2021 to 515 (67.9%) in 2023. Residential care peaked at 232 (28.1%) in 2022 before falling to 191 (25.2%) in 2023. Residential detox rose to 64 (7.7%) in 2022, then dropped to 50 (6.6%) in 2023. Short-term and long-term residential treatment followed similar trends. Hospital inpatient care remained stable, with total facilities decreasing from 80 (10%) in 2021 to 73 (9.6%) in 2023. Hospital inpatient detoxification increased slightly, while inpatient treatment rose from 47 (6%) in 2021 to 53 (7%) in 2023. Overall, facilities peaked at 826 in 2022 before declining to 759 in 2023.

Trends in clinical and therapeutic approaches

Over the years 2021 to 2023, the adoption of various clinical and therapeutic approaches for treating OUD and SUD demonstrated consistent trends. Substance Use Disorder Counseling remained the most widely utilized approach for both OUD and SUD. For OUD, usage rates were 724 (93.9%) in 2021, 739 (97.4%) in 2022, and 779 (94.3%) in 2023. Similarly, for SUD, the rates were 678 (87.9%) in 2021, 714 (94.1%) in 2022, and 745 (90.2%) in 2023. Table 4 presents the utilization of various clinical and therapeutic approaches in facilities treating OUD and other SUD from 2021 to 2023, highlighting the percentage and number of facilities adopting these methods.

**Table 4:** Clinical and therapeutic approaches for opioid and substance use disorders n(%): Number (percentage); - : None

Clinical/Therapeutic Approach	Opioid Use Disorder			Other Substances		
	2021 (n(%))	2022 (n(%))	2023 (n(%))	2021 n(%)	2022 (n(%))	2023 (n(%))
Substance use disorder counseling	724 (93.9%)	739 (97.4%)	779 (94.3%)	678 (87.9%)	714 (94.1%)	745 (90.2%)
12-step facilitation	339 (44%)	371 (44.9%)	371 (44.9%)	326 (42.3%)	363 (43.9%)	363 (43.9%)
Brief intervention	561 (72.8%)	622 (81.9%)	626 (75.8%)	533 (69.1%)	604 (79.6%)	606 (73.4%)
Cognitive behavior therapy	664 (86.1%)	704 (92.8%)	726 (87.9%)	632 (82%)	681 (89.7%)	706 (89.5%)
Contingency management/motivational incentives	298 (38.7%)	347 (45.7%)	348 (42.1%)	294 (38.1%)	342 (45.1%)	351 (42.5%)
Motivational interviewing	704 (91.3%)	730 (96.2%)	760 (92%)	669 (86.8%)	706 (93%)	729 (88.3%)
Trauma-related counseling	598 (77.6%)	662 (87.2%)	658 (79.7%)	574 (74.4%)	642 (84.6%)	636 (77%)
Anger management	503 (65.2%)	581 (76.5%)	570 (69%)	473 (61.3%)	570 (75.1%)	553 (66.9%)
Matrix model	204 (26.5%)	217 (28.6%)	234 (28.3%)	198 (25.7%)	210 (27.7%)	227 (27.5%)
Community reinforcement plus vouchers	47 (6.1%)	61 (8%)	69 (8.4%)	45 (5.8%)	56 (7.4%)	65 (7.9%)
Relapse prevention	696 (90.3%)	721 (95%)	761 (92.1%)	652 (84.6%)	693 (91.3%)	729 (88.3%)
Telemedicine/telehealth therapy	580 (75.2%)	617 (81.3%)	639 (77.4%)	545 (70.7%)	589 (77.6%)	605 (73.2%)
Other treatment approaches	104 (13.5%)	126 (16.6%)	122 (14.8%)	107 (13.9%)	128 (16.9%)	120 (14.5%)
None of the clinical/therapeutic approaches above are offered	—	—	—	—	—	1 (0.1%)

The implementation of 12-Step Facilitation remained stable, with 44% (339) of facilities offering it for OUD in 2021, 371 (44.9%) in 2022, and 371 (44.9%) in 2023. SUD facilities showed similar rates, at 326 (42.3%) in 2021, 363 (43.9%) in 2022, and 363 (43.9%) in 2023. Motivational Interviewing maintained high utilization, reaching 704 (91.3%) for OUD in 2021, 730 (96.2%) in 2022, and 760 (92%) in 2023, with SUD facilities showing similar trends. CBT remained widely used, with OUD facilities reporting 664 (86.1%) in 2021, 704 (92.8%) in 2022, and 726 (87.9%) in 2023. Trauma-related counseling remained prevalent but fluctuated. Telehealth Therapy grew in adoption but saw slight declines in 2023. Less common approaches, such as Community Reinforcement Plus Vouchers and the Matrix Model, remained below 30% across all years for both OUD and SUD. The data highlights the strong reliance on evidence-based approaches such as CBT, motivational interviewing, and substance use disorder counseling, alongside emerging trends like telehealth therapy.

Trends in the utilization of MAT and OTPs

Facilities with OTPs remained consistent, with a slight increase from 156 (8.4%) in 2021 to 172 (8.3%) in 2022 and 175 (8.4%) in 2023. Table 5 highlights the number and percentage of facilities offering OTPs, clients receiving MAT/MOUD at OTP and non-OTP facilities, and the breakdown by medication type across 2021-2023.

**Table 5:** Utilization of medication-assisted treatment (MAT) and opioid treatment programs (OTPs) n(%): Number (percentage); OTPs: Opioid treatment programs; MAT/MOUD: Medication-assisted treatment/Medications for opioid use disorder

Category	2021 (n(%))	2022 (n (%))	2023 (n(%))
Facilities with OTPs	156 (8.4%)	172 (8.3%)	175 (8.4%)
Clients receiving MAT/MOUD at OTP facilities			
Any MAT/MOUD	39,534 (9.3%)	39,595 (8.5%)	34,186 (7.8%)
Methadone	35,424 (9.6%)	37,328 (9.3%)	31,634 (8.4%)
Buprenorphine	3,835 (7.8%)	1,973 (3.5%)	2,049 (4.8%)
Naltrexone	275 (5.3%)	294 (5.1%)	503 (6.7%)
Clients receiving MAT/MOUD at non-OTP facilities			
Any MAT/MOUD	13,008 (6.4%)	16,757 (7.1%)	12,683 (4.8%)
Buprenorphine	10,733 (6%)	14,654 (7.3%)	10,238 (4.3%)
Naltrexone	2,275 (9.2%)	2,103 (6.3%)	2,445 (8.2%)

Clients receiving MAT/MOUD at OTP facilities declined from 39,534 (9.3%) in 2021 to 39,595 (8.5%) in 2022 and 34,186 (7.8%) in 2023. Methadone remained the primary treatment, with numbers dropping from 35,424 (9.6%) in 2021 to 37,328 (9.3%) in 2022 and 31,634 (8.4%) in 2023. Buprenorphine use fell sharply from 3,835 (7.8%) in 2021 to 1,973 (3.5%) in 2022, rising slightly to 2,049 (4.8%) in 2023. Naltrexone usage increased steadily. Non-OTP facilities showed fluctuating MAT/MOUD use, peaking in 2022 before declining in 2023. Methadone remained dominant, with shifting trends in buprenorphine and naltrexone use.

Trends in clients receiving MAT at OTP facilities

**Figure 2:**
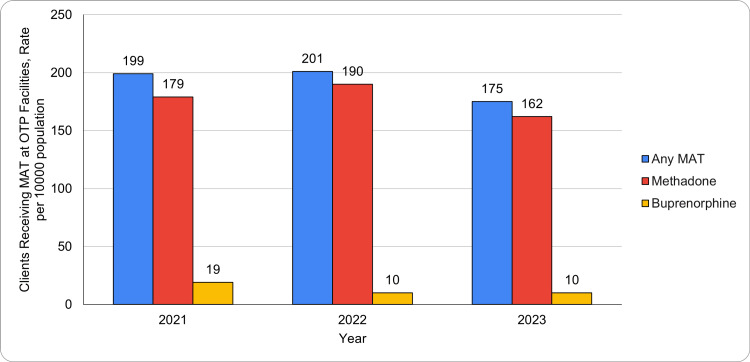
Clients receiving MAT at OTP facilities rate per 100,000 population OTP: Opioid treatment program; MAT: Medication-assisted treatment

The number of clients receiving any MAT at OTP facilities declined from 199 in 2021 to 201 in 2022 and then dropped to 175 in 2023. Methadone treatment was the most frequently used, with client numbers decreasing from 179 in 2021 to 190 in 2022, and falling further to 162 in 2023. Buprenorphine treatment showed a decline in 2022, dropping from 19 in 2021 to 10 in 2022, maintaining that number in 2023. Naltrexone usage saw a gradual increase, rising from 1 in 2021 to 3 in 2023, although it remained relatively low compared to other treatments. Figure 2 shows the annual trends in clients receiving MAT at OTP facilities per 100,000 population, with methadone being the most utilized treatment.

## Discussion

The analysis of Substance Use Treatment Services in New York (2021-2023), based on data from the N-SUMHSS, reveals important trends in service provision, client care, and treatment outcomes. These findings underscore the evolving nature of SUD treatment across the state, influenced by changes in service types, treatment modalities, and the evolving healthcare landscape. This discussion contextualizes these findings within broader trends in substance use treatment services and their implications for healthcare policy, access to care, and treatment efficacy in New York.

From 2021 to 2023, New York witnessed an overall increase in the number of facilities providing substance use disorder treatment, with a slight fluctuation across the years. The number of facilities with OTPs increased from 156 (8.4%) in 2021 to 175 (8.4%) in 2023. This growth indicates continued recognition of the importance of OTPs in the management of OUDs. Despite a small decline in the overall number of facilities in 2023 (759), the overall availability of treatment facilities remained relatively stable, suggesting a resilient system capable of adapting to fluctuating demand [10].

In particular, the rise in the proportion of private non-profit organizations offering treatment services, which accounted for a significant majority of the total facilities, highlights the essential role of non-profit entities in providing sustainable, community-based treatment options. The trend toward more non-profit treatment facilities may be indicative of their ability to tap into state funding and provide affordable services, which is critical in addressing the increasing demand for SUD treatment across New York. The dominance of private non-profits is particularly important when considering the state's strategy for expanding access to care for underprivileged and marginalized populations. The study by Amoaka et al. identified four distinct clusters of SUD treatment facilities in New York, varying in ownership, service comprehensiveness, and efforts to reduce barriers to care [11,12].

The availability and usage of MAT and MOUD are key indicators of the quality and accessibility of SUD treatment services. The data show a steady trend in the percentage of clients receiving MAT at OTP facilities, although there is a noticeable decline in the number of clients seeking MAT services from 39,534 (9.3%) in 2021 to 34,186 (7.8%) in 2023. This decline may reflect several factors, including shifts in opioid use patterns, changes in state or federal treatment protocols, or evolving client preferences [2,13].

While MAT at OTP facilities was primarily centered on methadone treatment, a notable decline in the use of methadone from 35,424 (9.6%) in 2021 to 31,634 (8.4%) in 2023 was observed. This drop in methadone use could be due to increasing awareness and access to alternative therapies, including buprenorphine, which experienced a modest increase in use over the same period. Buprenorphine’s appeal lies in its dual role as both a treatment for OUD and as a maintenance therapy option, providing greater flexibility in managing withdrawal symptoms and cravings [14]. Buprenorphine has also been shown to have less risk of overdose due to its ceiling effect, which makes it a better alternative for treatment and risk/harm reduction [14]. Also, its long-acting injection formulation (Sublocade) forms another basis for its preference, due to its proven benefits of a better clinical outcome [14-23].

Naltrexone, on the other hand, saw an uptick in its use at OTP facilities, which suggests a gradual shift toward incorporating long-acting medications and opioid-blocking treatments in the management of OUD. Despite naltrexone’s role as a promising treatment, its uptake remained comparatively lower than Methadone and Buprenorphine, highlighting the challenges in widespread implementation and patient adherence, especially given the need for patients to remain opioid-free before initiating Naltrexone therapy [15].

In contrast, MAT at non-OTP facilities showed significant variations in both the number of clients served and the types of MAT used. Buprenorphine treatment remained the dominant choice, suggesting that non-OTP facilities are increasingly integral in supporting clients with OUD, particularly through outpatient services. The increase in the number of clients receiving MAT at non-OTP facilities from 13,008 (6.4%) in 2021 to 16,757 (7.1%) in 2022, followed by a slight decrease in 2023, reflects the growing recognition of the role of outpatient and community-based treatment in managing SUD [2].

The broader range of clinical and therapeutic approaches adopted for treating substance use disorders in New York reflects an evolving treatment landscape. The frequent use of substance use disorder counseling, CBT, and motivational interviewing across both opioid and non-opioid use disorders underscores the critical role of psychotherapy in complementing pharmacological treatment. Substance use disorder counseling was utilized by 93.9% of clients in 2021 for OUD and 87.9% for other substances, reflecting a commitment to holistic, integrated care. Additionally, the widespread adoption of CBT further supports the emphasis on addressing underlying behavioral patterns and maladaptive thinking that contribute to addiction [16].

Other therapeutic approaches, such as contingency management, trauma-related counseling, and anger management, also demonstrated increased implementation over time. The growth in the use of motivational interviewing, which increased from 91.3% in 2021 to 92% in 2023 for OUD, suggests a deeper integration of patient-centered, empathetic treatment approaches aimed at fostering patient autonomy and self-efficacy in recovery. Similarly, trauma-related counseling, essential for addressing co-occurring mental health conditions, expanded from 77.6% in 2021 to 79.7% in 2023 for OUD clients, reflecting the state's recognition of the interconnectedness between substance use disorders and trauma [17].

Telemedicine emerged as a prominent treatment modality, with significant increases in its use for both opioid and non-opioid substance use disorders. From 2021 to 2023, telemedicine use increased across both populations, demonstrating the effectiveness and scalability of virtual care, particularly during the COVID-19 pandemic. Telemedicine’s potential in overcoming geographical barriers and improving access to care for rural and underserved populations makes it a vital component of New York’s substance use treatment framework [18]. The study by Harris et al. reported that high-performing providers emphasized destigmatizing MOUD, addressing systemic barriers, and enhancing care integration through multidisciplinary approaches, community partnerships, telehealth, and harm reduction strategies [19,20].

Overall, the data from 2021 to 2023 reflect substantial growth and adaptation in New York's substance use treatment services [21]. The increase in the number of facilities offering a range of MAT options, the expansion of non-profit organizations, and the incorporation of evidence-based therapeutic modalities indicate a robust and responsive treatment infrastructure. However, the decline in methadone use and the variability in MAT uptake suggest that challenges remain in fully addressing the diverse needs of individuals with substance use disorders [22]. Still, it is noteworthy that, while the increase in buprenorphine use may be attributed to its safety profile, better evidence of treatment adherence and treatment success, policy adjustments may need to be discussed to accommodate physician's ability to prescribe the medication for opioid use disorder treatment. Also, while there may be identifiable challenges with tapering or discontinuing Buprenorphine or its combination formulation, further studies need to be done to better explore long-term treatment expectations and provide effective solutions to manage existing challenges of substance use disorders treatments with these opioid agonists [22].

Future directions should focus on expanding access to MAT in underserved areas, increasing the use of telemedicine, and further integrating mental health and substance use services. Continued monitoring and adaptation of treatment strategies will be crucial in responding to emerging trends in substance use and ensuring that New York’s substance use treatment services can effectively meet the needs of all its residents.

Strengths and limitations

One of the primary strengths of this analysis lies in its use of comprehensive, statewide data from the N-SUMHSS, which provides valuable insights into trends in substance use treatment over three years (2021-2023). The data covers a wide range of treatment modalities, including MAT, therapeutic approaches, and facility types, offering a detailed understanding of service provision across New York. However, the analysis is limited by the reliance on self-reported data, which may be subject to reporting bias. Additionally, regional variations and other contextual factors, such as socioeconomic disparities, are not fully explored. The data does not provide in-depth insights into patient outcomes, making it challenging to assess the long-term effectiveness of the treatment strategies employed. Furthermore, the absence of inferential statistical testing limits our ability to draw causal conclusions; potential confounding factors (e.g., policy changes or socioeconomic differences) may influence observed trends; and the lack of sub‑state or regional analyses could mask important local variations. Future studies should incorporate outcome data and more granular geographic analysis for a deeper understanding of the service landscape.

## Conclusions

This analysis provides a comprehensive overview of the trends in substance use treatment services in New York over the past three years. The steady growth in treatment facilities, coupled with the increasing use of MAT and holistic therapeutic approaches, reflects the state’s commitment to expanding access to care for individuals with substance use disorders. These findings are indicative of evolving treatment patterns; future longitudinal and outcome‑based research is needed to confirm these trends. However, challenges remain, particularly regarding the decline in methadone use and the variability in treatment uptake across different regions. Moving forward, the integration of telemedicine, the expansion of MAT availability, and enhanced focus on co-occurring mental health conditions will be essential to meet the evolving needs of the population. The findings underscore the need for ongoing evaluation and adaptation of treatment strategies to ensure the continued effectiveness and accessibility of services.
